# BOLD data representing activation and connectivity for rare no-go versus frequent go cues

**DOI:** 10.1016/j.dib.2016.02.011

**Published:** 2016-02-09

**Authors:** Harma Meffert, Soonjo Hwang, Zachary T. Nolan, Gang Chen, James R. Blair

**Affiliations:** National Institutes of Health, United States

**Keywords:** Cognitive control, Go/No-go, fMRI, Generalized psychophysiological interactions, Inhibition

## Abstract

The neural circuitry underlying response control is often studied using go/no-go tasks, in which participants are required to respond as fast as possible to go cues and withhold from responding to no-go stimuli. In the current task, response control was studied using a fully counterbalanced design in which blocks with a low frequency of no-go cues (75% go, 25% no-go) were alternated with blocks with a low frequency of go cues (25% go, 75% no-go); see also “Segregating attention from response control when performing a motor inhibition task: Segregating attention from response control” [1]. We applied a whole brain corrected, paired *t*-test to the data assessing for regions differentially activated by low frequency no-go cues relative to high frequency go cues. In addition, we conducted a generalized psychophysiological interaction analysis on the data using a right inferior frontal gyrus seed region. This region was identified through the BOLD response *t*-test and was chosen because right inferior gyrus is highly implicated in response inhibition.

## Specification table

**Table:** 

Subject area	Cognitive Neuroscience
More specific subject area	Response control
Type of data	Nifti
How data was acquired	3 T Functional Magnetic Resonance Imaging
Data format	Analyzed
Experimental factors	Go cues and no-go cues were visually presented to participants with a factorial design, in which blocks with a low frequency of no-go cues (75% go, 25% no-go) were alternated with blocks with a low frequency of go cues (25% go, 75% no-go).
Experimental features	Event-related Go/No-go task using functional Magnetic Resonance Imaging in healthy adults.
Data source location	Bethesda, MD, United States of America
Data accessibility	With this article

## Value of the date

• These data could inform researchers with an interest in cognitive control, particularly response inhibition.
• Data show differential BOLD responses and connectivity with right inferior gyrus to infrequent no-go versus frequent go trials.
• Data show that infrequent no-go cues recruit bilateral inferior frontal gyrus more strongly compared to frequent go cues.
• Data show differential connectivity with regions such as the anterior insula and pre-Supplementary Motor cortex.
• The data can be used to further outline the functional characteristics of the response inhibition network featuring the right inferior frontal gyrus.

## 1 Data

Healthy adult participants performed a rapid event-related Go/No-go task, while undergoing functional Magnetic Resonance Imaging. In this task, blocks with a low frequency of no-go cues (75% go, 25% no-go) were alternated with blocks with a low frequency of go cues (25% go, 75% no-go). This data has previously been analyzed within the context of a fully counterbalanced design [1]. The current manuscript presents further analyses of these data. Table 1 reports results from a paired samples *t*-test (low frequency no-go cues versus high frequency go cues). Table 2 reports results from a generalized psychophysiological interaction [gPPI, [2]] using the right inferior frontal gyrus as seed region.

## 2 Experimental design, materials and methods

### 2.1 Participants

Twenty-two, right handed healthy adult volunteers (54.50% female; average age 25.95±4.54) consented to participate in the study. IQ was assessed with the Wechsler Abbreviated Scale of Intelligence [two-subtest, ([5]. Wechsler Abbreviated Scale of Intelligence)]; average IQ=123.18 (SD=11.63). For a fuller description, see [1].

### 2.2 Experimental design

Each trial began with the presentation of a picture of a go cue (Spiderman) or a no-go cue (Green Goblin) for 500 ms, followed by a jittered interval (1000–1500 ms) during which a fixation cross was presented. There were six different Spiderman and Green Goblin images. Participants were instructed to press the button as fast as possible whenever they saw a Go (Spiderman) cue. Participants had to respond within 1000 ms after target onset, otherwise the trial was recorded as a missed trial.

Trials were randomized within two types of blocks: High No-go Frequency context blocks (25% go cues and 75% no-go cues) and High Go Frequency context blocks (75% go cues and 25% no-go cues). Each block contained 72 trials. After each block, a fixation block was presented for 30 s. Each run contained 2 blocks, a High Go Frequency and a High No-go Frequency block, and took about 5.5 min. The order of frequency blocks within each run was counterbalanced across runs and participants. Participants completed four runs in total.

Stimuli were presented using Presentation (http://www.neurobs.com/).

### 2.3 Image acquisition

For full details see “1. Segregating attention from response control when performing a motor inhibition task: Segregating attention from response control”.

### 2.4 Image processing

Data were analyzed within the framework of the general linear model using Analysis of Functional Neuroimages ([6]. AFNI: software for analysis and visualization of functional magnetic resonance neuroimages). For full details see “1. Segregating attention from response control when performing a motor inhibition task: Segregating attention from response control”. The individuals’ functional EPI data were registered to the Talairach and Tournoux atlas ([7]. Co-planar stereotaxix atlas of the human brain).

The following 5 regressors were generated: indicator functions for go cues during a Low Go Frequency block, go cues during a High Go Frequency block, no-go cues during a Low No-go Frequency block, no-go cues during a High No-go Frequency block and incorrect responses.

### 2.5 Group level analysis on activation data

We performed a whole brain paired *t*-test, assessing for regions differentially activated by low frequency no-go cues compared to high frequency go cues (the contrast most often reported within the go/no-go literature).

### 2.6 Psychophysiological interactions

A generalized psychophysiological interaction ([2]. A generalized form of context-dependent psychophysiological interactions (gPPI): A comparison to standard approaches) analysis was conducted to examine differential task-based functional connectivity with right inferior frontal gyrus (see Table 1, region A). This region was chosen as a seed region because of widespread agreement for its importance in response inhibition. The average BOLD response in this seed region was extracted from the de-spiked, slice-time corrected, motion correction, spatially normalized time-series data. The seed time-series was first detrended and deconvolved. Four interaction terms were created by multiplying the detrended and deconvolved seed time-series with four indicator regressors, which indicated the onset of go cues during a Low Go Frequency block, go cues during a High Go Frequency block, no-go cues during a Low No-go Frequency block, no-go cues during a High No-go Frequency block. Finally, these four interaction terms were convolved with the hemodynamic response function to create four gPPI regressors per seed region. Linear regression modeling was performed using five task regressors (High Frequency Go, High Frequency No-go, Low Frequency Go, Low Frequency No-go and Incorrect trials), six motion regressors, a regressor reflecting the seed time-series, the four gPPI regressors and regressors to model a first-order baseline drift function. This produced a *β* coefficient and associated *t* statistic for each voxel and regressor.

We then conducted a whole brain group analysis on the first level gPPI contrasts using a 2 (Response type: Go or No-go) by 2 (Frequency: Low or High) repeated measures ANOVA. Data are reported at *p*=0.005 uncorrected, cluster size of 10 voxels. This threshold has shown to hold a good balance between Type I and Type II error rates Type ([8]. I and Type II error concerns in fMRI research: re-balancing the scale).

The data for the paired-samples *t*-test on low frequency no-go trials versus high frequency go trials is presented in Table 1. The data for the 2 (Response type: Go or No-go) by 2 (Frequency: Low or High) repeated measures ANOVA was conducted on the gPPI parameter estimates using, as a seed, the region of right IFG showing increased responses to no-go relative to go cues, is presented in Table 2.

## Figures and Tables

**Table 1:** Low frequency No-go versus High Frequency Go**.** Shown are the data from a paired samples *t*-test, contrasting Low Frequency No-go trials with High Frequency Go trials. All data are thresholded at *p*=0.005 uncorrected and a cluster extent threshold of 39 voxels (corresponding to map-wise false-positive probability of *p*<0.05). The last column indicates the pair-wise post-hoc *t*-tests. T=Target, N=Non-target.

Region^a^	Hemisphere	Brodmann’s area	*T*	Coordinates of peak significance (*x y z* )			Voxels	Post-hoc
Inferior frontal/middle frontal gyrus (A)	Right	44	4.483	28.5	16.5	26.5	259	N>G
Inferior frontal/middle frontal gyrus	Left	44	4.1303	−37.5	10.5	29.5	65	N>G
Precentral/postcentral gyrus	Left	4/3	−5.7096	−34.5	−25.5	50.5	230	G>N
Inferior parietal lobule	Right	40	5.3104	37.5	−37.5	41.5	217	N>G
Middle occipital gyrus	Right	19	5.5346	31.5	−76.5	11.5	450	N>G
Fusiform gyrus	Right	37/20	6.5461	28.5	−52.5	−9.5	287	N>G
Fusiform gyrus	Left	37/20	6.7791	−31.5	−46.5	−15.5	235	N>G
Cerebellum	Right	—	−6.5992	16.5	−49.5	−18.5	130	G>N
Precuneus	Left	31/7	4.3268	−25.5	−64.5	26.5	92	N>G

a The regions are according to the Talairach Daemon atlas (http://www.nitrc.org/projects/tal-daemon).

**Table 2:** gPPI data with right IFG from paired-samples *t*-test. Shown are the data of a 2 (Response type: Go or No-go) by 2 (Frequency: Low or High) repeated measures ANOVA on the gPPI *β* coefficients using the right IFG as seed region. All data are thresholded at *p*=0.005 uncorrected and a cluster extent threshold of 10 voxels. The last column indicates the pair-wise post-hoc *t*-tests. G=Go, N=No-go, L=Low, H=High.

Region^a^	Hemisphere	Brodmann’s area	*F*	Coordinates of peak significance (*x y z*)			Voxels	Post-hoc
**(a) Main effect of frequency**								
Anterior insula (ventral)	Left	13	33.12	−31.5	16.5	−9.5	34	L>H
Superior frontal gyrus	Right	10	18.44	31.5	49.5	17.5	17	L>H
Middle occipital gyrus	Left		20.06	−34.5	−76.5	−6.5	13	L>H
Middle temporal gyrus	Left		26.71	−61.5	−49.5	8.5	13	L>H
Cerebellum	Left	–	18.28	−40.5	−58.5	−27.5	15	L>H

**(b) Main effect of response type**								
Inferior frontal gyrus	Right	44	22.8	55.5	7.5	20.5	12	G>N
pre-Supplementary Motor Cortex	Right	6	20.87	10.5	−1.5	56.5	19	G>N
Hippocampus/parahippocampal gyrus	Left	35	19.35	−31.5	−28.5	−6.5	71	N>G
Hippocampus/parahippocampal gyrus	Right	35	20.7	31.5	−25.5	−9.5	60	N>G
Parahippocampal gyrus	Left	36	25.16	−34.5	−19.5	−21.5	62	N>G
Amygdala	Right	–	20.07	31.5	−4.5	−9.5	52	N>G
Amygdala/putamen	Left	–	17.56	−28.5	1.5	−6.5	38	N>G
Supramarginal gyrus	Right	40	18.75	55.5	-37.5	32.5	49	G>N
Fusiform gyrus	Left	37	14.51	−43.5	−43.5	−15.5	21	N>G
Superior temporal gyrus	Right	21	14.62	40.5	4.5	−24.5	13	N>G
Inferior temporal gyrus	Right	20	15.26	52.5	−7.5	−21.5	11	N>G

**(c) Interaction between response type and frequency**								
Middle cingulate gyrus	Left	24	27.98	−4.5	4.5	29.5	30	
Postcentral gyrus	Left	4	19.38	−46.5	−16.5	29.5	42	
Postcentral gyrus	Right	4	30.39	43.5	−16.5	35.5	29	
Parahippocampal gyrus	Left	36	30.43	−28.5	−19.5	−21.5	19	
Hippocampus/amygdala	Right	–	16.51	34.5	−25.5	−9.5	13	
Hippocampus	Right	–	17.5	22.5	−13.5	−15.5	11	
Fusiform gyrus	Left	37	15.62	−46.5	−46.5	−15.5	16	
Cuneus	Right	18	22.04	1.5	−85.5	32.5	33	
Cuneus	Right	17	13.02	1.5	−88.5	8.5	16	

a The regions are according to the Talairach Daemon atlas (http://www.nitrc.org/projects/tal-daemon).
